# No Association between Arsenic Exposure from Drinking Water and Diabetes Mellitus: A Cross-Sectional Study in Bangladesh

**DOI:** 10.1289/ehp.0901559

**Published:** 2010-05-13

**Authors:** Yu Chen, Habibul Ahsan, Vesna Slavkovich, Gretchen Loeffler Peltier, Rebecca T. Gluskin, Faruque Parvez, Xinhua Liu, Joseph H. Graziano

**Affiliations:** 1 Department of Environmental Medicine, New York University School of Medicine, New York, New York, USA; 2 Department of Health Studies; 3 Department of Medicine; 4 Department of Human Genetics and; 5 Cancer Research Center, The University of Chicago, Chicago, Illinois, USA; 6 Department of Environmental Health Sciences, Mailman School of Public Health, Columbia University, New York City, NY, USA

**Keywords:** arsenic exposure, Bangladesh, cross-sectional studies, diabetes, environmental epidemiology

## Abstract

**Background:**

The long-term effects of arsenic exposure from drinking water at levels < 300 μg/L and the risk of diabetes mellitus remains a controversial topic.

**Method:**

We conducted a population-based cross-sectional study using baseline data from 11,319 participants in the Health Effects of Arsenic Longitudinal Study in Araihazar, Bangladesh, to evaluate the associations of well water arsenic and total urinary arsenic concentration and the prevalence of diabetes mellitus and glucosuria. We also assessed the concentrations of well water arsenic, total urinary arsenic, and urinary arsenic metabolites in relation to blood glycosylated hemoglobin (HbA1c) levels in subsets of the study population.

**Results:**

More than 90% of the cohort members were exposed to drinking water with arsenic concentration < 300 μg/L. We found no association between arsenic exposure and the prevalence of diabetes. The adjusted odds ratios for diabetes were 1.00 (referent), 1.35 [95% confidence interval (CI), 0.90–2.02], 1.24 (0.82–1.87), 0.96 (0.62–1.49), and 1.11 (0.73–1.69) in relation to quintiles of time-weighted water arsenic concentrations of 0.1–8, 8–41, 41–91, 92–176, and ≥ 177 μg/L, respectively, and 1.00 (referent), 1.29 (0.87–1.91), 1.05 (0.69–1.59), 0.94 (0.61–1.44), and 0.93 (0.59–1.45) in relation to quintiles of urinary arsenic concentrations of 1–36, 37–66, 67–114, 115–204, and ≥ 205 μg/L, respectively. We observed no association between arsenic exposure and prevalence of glucosuria and no evidence of an association between well water arsenic, total urinary arsenic, or the composition of urinary arsenic metabolites and HbA1c level.

**Conclusions:**

Our findings do not support an association of arsenic exposure from drinking water and a significantly increased risk of diabetes mellitus in the range of levels observed. Further prospective studies would be valuable in confirming the findings.

Arsenic is abundant in the earth’s crust and can be released to groundwater under certain conditions. It has been estimated that 13 million Americans have been exposed to public water supplies with 10–50 μg/L arsenic ([Bibr b55-ehp-118-1299][Bibr b56-ehp-118-1299]). In Bangladesh, more than 50 million people have been chronically exposed to drinking groundwater with arsenic concentrations exceeding the World Health Organization standard (10 μg/L) (British Geological Survey 1999). In many parts of the world where groundwater is an important source of drinking water, exposure to inorganic arsenic (InAs) from drinking water has been linked to increased risks of skin cancer (Hsueh et al. 1997), internal cancers such as bladder, lung, and liver cancers (Chen et al. 1988, 2004a), and cardiovascular disease (Chen et al. 1995, 1996). In addition, systematic reviews of the literature on the association between arsenic exposure and diabetes mellitus suggest a possible role of high arsenic exposure (> 500 μg/L) in diabetes mellitus (Chen et al. 2007; Navas-Acien et al. 2006).

However, the effects of exposure to lower concentrations of arsenic on diabetes are unclear. In a cross-sectional study of 1,185 residents in Wisconsin, Zierold et al. (2004) calculated odds ratios (ORs) for diabetes of 1.4 [95% confidence interval (CI), 0.8–2.3] and 1.1 (95% CI, 0.5–2.2) for arsenic exposure levels of 2–10 μg/L and > 10 μg/L, respectively, with the referent group < 2 μg/L. Compared with that of the general population in Utah, Lewis et al. (1999) found that the standard mortality ratio for diabetes was not elevated among members of a Mormon community in Millard County, Utah, with < 200 μg/L of arsenic in drinking water. In a case-control study in Mexico, the ORs for diabetes were 1.9 (95% CI, 1.1–3.4) and 2.7 (95% CI, 1.5–4.6) for groups with total urinary arsenic of 64–104 μg/L and > 104 μg/L, respectively (Coronado-Gonzalez et al. 2007). More recently, in a cross-sectional study from National Health and Nutrition Examination Survey (NHANES), Navas-Acien et al. (2008) reported that the OR for diabetes was 3.6 (95% CI, 1.2–10.8) when they compared participants at the 80th percentile with those at the 20th percentile for urinary arsenic (16.5 vs. 3.0 μg As/L). Several reanalyses of the same data, as well as updated data with more subjects from NHANES, suggest that opposing results may be explained by differences in how urinary creatinine and arsenobetaine (AsB) in the urine were handled in the statistical modeling (Longnecker 2009; Navas-Acien et al. 2009; Steinmaus et al. 2009a, 2009b). Given this controversy, studies in populations with long-term arsenic exposure at 10–300 μg/L are important in justifying future studies of arsenic exposure at lower levels.

In addition, to better understand the pathophysiology and mechanism by which arsenic exposure may lead to diabetes, studies of biomarkers for manifestations of diabetes are needed. Glycosylated hemoglobin (HbA1c) is the result of the nonenzymatic glycosylation of hemoglobin, which reflects the integrated blood glucose level during the preceding 3–4 months; to meaure HbA1c, fasting is not necessary. Although several studies have suggested that suboptimal arsenic methylation capacity, which was indicated by a relatively high proportion of monomethylarsonic acid (MMA) in urine, is positively associated with the risk for health effects of arsenic exposure such as the risk of skin and bladder cancer (Hsueh et al. 1997; Kopp 2005; Yu et al. 2000), the relationship between arsenic methylation capacity and HbA1c is unknown.

We established a study of 11,746 individuals in Araihazar, Bangladesh, in the year 2000 to assess arsenic-related health effects. We conducted cross-sectional analyses to evaluate the associations of arsenic exposure with diabetes status and glucosuria. In a subset of 2,100 participants, we also assessed the relationship of arsenic exposure and the composition of urinary arsenic metabolites with HbA1c levels.

## Methods

### The Health Effects of Arsenic Longitudinal Study (HEALS)

The parent study HEALS is an ongoing prospective cohort study in Araihazar, Bangladesh. Details of the study methodologies have been presented elsewhere (Ahsan et al. 2006a; Parvez et al. 2006). Prior to subject recruitment, water samples and their geographic coordinates were collected for 5,966 contiguous wells in a well-defined geographic area of 25 km^2^ in Araihazar. Between October 2000 and May 2002, 11,746 men and women ≥ 18 years of age were recruited, with a response rate of 97.5% (Ahsan et al. 2006a). Verbal informed consent was obtained from study participants. The study procedures were approved by the Columbia University Institutional Review Board and the Ethical Committee of the Bangladesh Medical Research Council.

At baseline recruitment, lifestyle and demographic information were collected using structured interviews. Trained study physicians measured height and weight using a locally manufactured tape measure and a Misaki (Okaka, Japan) scale (calibrated weekly), respectively. Both height and weight were measured three times at baseline and averaged. Venous whole blood samples were collected for 91.8% of the overall 11,746 cohort participants in 3-mL vacutainers containing EDTA as anticoagulant. A spot urine sample was collected in 50-mL acid-washed tubes for 95.6% of the cohort participants. Both blood and urine samples were kept in portable coolers immediately after collection and were processed within 2–8 hr and transferred to −20°C freezers before being shipped to Columbia University on dry ice within 1–2 months.

### Measurements of arsenic exposure

At baseline, water samples from all 5,966 tube wells in the study area were collected in 50-mL acid-washed tubes after well pumping for 5 min (van Geen et al. 2002, 2003). Total arsenic concentration was determined by graphite furnace atomic-absorption spectrometry (GFAA) with a Hitachi Z-8200 system (Hitachi Corp., Tokyo, Japan) (van Geen et al. 2002). Samples that fell below the detection limit of GFAA (5 μg/L) were subsequently analyzed by inductively coupled plasma mass spectrometry (ICP-MS), with a detection limit of 0.1 μg/L (Cheng et al. 2005). All participants were primary users of one of the 5,966 tube wells, designated as the “index” well, for at least 3 years. We derived a time-weighted arsenic concentration (TWA) as a function of drinking durations and well arsenic concentrations [TWA (micrograms per liter) = ∑ *C_i_T_i_*/∑ *T_i_*, where *C_i_* and *T_i_* denote the well arsenic concentration and drinking duration for the *i*th well] (Ahsan et al. 2006b). The average duration of well use for wells with a known arsenic concentration accounted for 25% of lifetime for both sexes (Ahsan et al. 2006b).

Total urinary arsenic concentration was measured by GFAA, using a Perkin-Elmer AAnalyst 600 graphite furnace system (PerkinElmer, Waltham, MA, USA) with a detection limit of 2 μg/L, as previously described (Nixon et al. 1991). Urinary creatinine was analyzed using a method based on the Jaffe reaction for adjusting urinary total arsenic concentration (Slot 1965). All the urine samples were detectable for total urinary arsenic.

As part of case–control and case–cohort studies of other health outcomes (Ahsan et al. 2007), urinary arsenic metabolites were measured in a random 10% of HEALS participants using a method described by Reuter et al. (2003). This method employs HPLC separation of AsB, arsenocholine (AsC), As^V^, As^III^, MMA, and dimethylarsinic acid (DMA), followed by detection by ICP-MS with dynamic reaction cell (ICP-MS-DRC), with a detection limit of 0.2 μg/L for AsB and AsC and of 0.1 μg/L for all other metabolites. Arsenic methylation indices including the percentage of InAs, MMA, and DMA of the total urinary arsenic were calculated after subtracting AsB and AsC (i.e., nontoxic organic arsenic from dietary sources) from the total. We also constructed two methylation indices: primary methylation index (PMI), that is, the ratio of MMA to InAs and the secondary methylation index (SMI), that is, the ratio of DMA to MMA.

### Measurement of dietary intakes

Dietary intakes were measured at baseline with a validated semiquantitative food frequency questionnaire (FFQ) designed for the study population. Detailed information on the design and the validation of the FFQ has been published elsewhere (Chen et al. 2004b). The results of the validation study indicate that the FFQ can provide reasonably valid measurements for long-term dietary intakes of common foods, macronutrients, and common micronutrients (Chen et al. 2004b).

### Diabetes-related outcomes

This study is a cross-sectional analysis using the baseline data of the HEALS. At baseline, interviewers recorded generic names of all medicines that the study participants were taking regularly. At the first 2-year follow-up, participants were asked if they had ever been diagnosed by a physician with diabetes, and date of diagnosis was ascertained for those who gave a positive answer. The study physicians and interviewers were blinded to urinary arsenic and well water arsenic levels (Ahsan et al. 2006b). Because baseline interviews did not include questions on diabetes status, we retrospectively identified participants who had a self-reported physician diagnosis of diabetes prior to baseline. Those with a date of diagnosis earlier than the baseline interview date were defined as prevalent cases; cases diagnosed between baseline and first follow-up visit (*n* = 37) were excluded from the study. All the regular users of insulin or oral hypoglycemic medication reported that they had a physician diagnosis of diabetes. All the participants < 20 years old at baseline were excluded to focus on type 2 diabetes as the disease of interest.

Blood samples from the first 2,100 consecutively recruited participants were analyzed for HbA1c. The percentage of HbA1c was measured by the Helena Glyco-Tek affinity column method (Helena, Beaumont, TX, USA) using a Lambda UV/ViS spectrophotometer (PerkinElmer). This is an affinity microchromatographic methodology that quantitates all glycated hemoglobin, including the A1c fraction, and is not subject to interference by labile glycated hemoglobin (Klenk et al. 1982). The HbA1c value was determined by comparing the two solutions using a spectrophotometer operating at 415 nm.

At baseline, dipstick urinalysis was performed by a trained physician on freshly evacuated spot urine samples using the Chemstrip Micral Test Strips (Roche Diagnostics, Indianapolis, IN, USA). The results of the urine test were based on a color scale that quantified glucosuria as negative, 50 mg/dL, 100 mg/dL, 200 mg/dL, 500 mg/dL, and 1,000 mg/dL.

### Statistical analysis

We first conducted descriptive analyses comparing participants with diabetes and those without diabetes at baseline in terms of sociodemographic characteristics, established risk factors of type 2 diabetes, dipstick glucosuria, and HbA1c levels. We estimated ORs for diabetes and for glucosuria in relation to quintiles of arsenic exposure variables using unconditional logistic regression. Because treatments for diabetes may influence glucosuria status, participants with diabetes were excluded from those who tested negative for glucosuria in estimating ORs for glucosuria (*n* = 90). Established risk factors of diabetes that may influence health effects of arsenic as indicated in previous studies (Ahsan et al. 2006b; Argos et al. 2007; Chen et al. 2006) were considered as potential confounders. The associations between potential confounders and arsenic exposure variables are shown in Supplemental Material, Table 1 (doi:10.1289/ehp.0901559). We first adjusted for age (years), sex (men and women), and body mass index (BMI; kilograms per meter squared), and in a separate model we additionally adjusted for smoking status (never, past, and current), and educational attainment (years). To evaluate the extent by which the association between arsenic exposure and diabetes could be attributable to arsenic content in foods, we also adjusted the ORs for intakes of fish and rice (grams per day) because fish can accumulate organic arsenic, which may influence total urinary arsenic concentration, and because rice may contain InAs from the soil (Das et al. 2004). In addition, Pearson correlation was used to evaluate the relative validity of total urinary arsenic and urinary metabolites in assessing well water arsenic exposure and the extent to which fish and rice intakes contribute to total urinary arsenic.

Because BMI is a strong risk factor for diabetes, we conducted stratification analysis to evaluate the associations of arsenic exposure with diabetes and glucosuria in high and low BMI category, which was defined by the median value in the overall population. Significance of multiplicative interaction between BMI and arsenic exposure was determined by the *p*-values associated with cross-product terms for the dichotomized BMI and quintiles of arsenic exposure expressed as an ordinal variable in multivariate logistic models. Additional stratification was conducted for age and sex.

In addition, we estimated the ratio of geometric means of arsenic exposure comparing diabetics and nondiabetics using linear regression models on log-transformed arsenic levels. Sensitivity analyses were conducted to estimate the ratio of geometric means of arsenic exposure comparing the presence of both diabetes and glucosuria with absence of both conditions.

Arsenic concentrations measured in spot urine samples may be affected by the variation in dilution due to variation in the state of hydration. However, prevalent cases of diabetes have a lower level of urinary creatinine compared with noncases (Barr et al. 2005; de Fine et al. 2006), because they may have been hypercatabolic for a long period, which results in a smaller muscle mass and therefore diminished urinary creatinine (de Fine et al. 2006). Diabetes-related hyperfiltration could also lead to a low level of creatinine and an increased urine volume (Mogensen 1994), which would result in differences in urinary creatinine-adjusted arsenic concentrations. Adjustment for factors affected by the disease may lead to bias away from the null in epidemiologic studies and therefore is not recommended (Greenland 2003). Nevertheless, because adjusting urinary arsenic concentration for the excretion of urinary creatinine is a common way of correcting the variation in dilution (Nermell et al. 2008), we show results with and without adjustment for urinary creatinine in separate models.

To evaluate the association between arsenic exposure and HbA1c concentration, we computed least squares means of HbA1c level according to arsenic exposure categories using linear regression. Model construction procedures were similar to those for logistic regression. The relationship between urinary arsenic methylation indices and HbA1c was assessed in 368 subjects who were part of both the 10% random selection of the overall study population (for whom urinary arsenic metabolites were measured) and the first 2,100 enrolled subjects (who were tested for HbA1c). Results in the analyses of HbA1c excluding those with diabetes (*n* = 45) were similar and therefore are not shown.

In all analyses, controlling or excluding cases with skin lesions did not materially affect the results, and therefore these results are not shown. All analyses were peformed using SAS 9.1.3 (SAS Institute Inc., Cary, NC, USA). All tests were two sided, and *p* < 0.05 was considered significant.

## Results

Participants who reported a diagnosis of diabetes prior to baseline (*n* = 241) were older and more likely to be past cigarette smokers, have higher blood pressure and lower levels of urinary creatinine, and have higher levels of BMI, urinary glucose, and HbA1c than were those who did not report a diagnosis of diabetes (*n* = 11,078) (Table 1). Only 1% of the individuals without diabetes tested positive on urinary glucose, whereas 61% of the individuals with diabetes tested positive (*p* < 0.01). In a subgroup of 2,100 participants, the median of HbA1c level was 6.8% in participants with diabetes, substantially higher than the median level among participants without diabetes (4.9%, *p* < 0.01). Diabetes was also positively related to betel nut use and indices of socioeconomic status in rural Bangladesh including educational attainment, TV ownership, and land ownership (*p* < 0.01). These data were consistent with studies in rural areas in Bangladesh (Abu et al. 1995, 1997; Sayeed et al. 2003). Bivariate analysis also shows that the medians of well water arsenic and urinary arsenic concentration were slightly higher in noncases of diabetes compared with cases (62.0 μg/L vs. 53.0 μg/L for well water arsenic and 45.9 μg/L vs. 44.1 μg/L for urinary arsenic, respectively).

In the overall analysis, we found no evidence for an association between either TWA or urinary arsenic concentration and the prevalence of diabetes (Table 2). Multivariate adjustment for potential confounders had little impact on the effect estimates, although associations between urinary arsenic and diabetes prevalence were stronger (but not significant) after adjusting for urinary creatinine (Model 3). Associations also were not evident when the analysis was restricted to participants known to have more than 4 years of exposure (average = 11.2 years) from the well used to determine their TWA [Supplemental Material, Table 2 (doi:10.1289/ehp.0901559)]. No association was found between arsenic exposure and diabetes prevalence within categories of BMI, although the number of cases among those with low BMI (< 20, *n* = 67–71) was limited. Stratification by age (< 35 years and ≥ 35 years at baseline) and sex also did not suggest any subgroup-specific associations [Supplemental Material, Table 2 (doi:10.1289/ehp.0901559)].

The correlation of well water arsenic with total urinary arsenic, DMA, and MMA concentration was 0.70, 0.61, and 0.57, respectively. These results indicate a good relative validity of urinary arsenic as a reflection of arsenic exposure from well water. The correlation between rice intake and total urinary arsenic was 0.03 and 0.04 among the overall study population and in those with baseline well water arsenic < 50 μg/L, respectively. Among the 10% random sample of the cohort with data on urinary arsenic metabolites, the correlation between rice intake and urinary DMA concentration was 0.01 and 0.02 among the overall and those with baseline well water arsenic < 50 μg/L, respectively. The correlation of total urinary arsenic concentration with urinary AsB and AsC concentration were 0.13 and 0.06, respectively. These data suggest that rice and seafood intakes contributed very little to total urinary arsenic and urinary DMA concentration in the study population. Effect estimates of ORs for diabetes in relation to arsenic exposure did not change appreciably with additional adjustment for fish and rice intake (data not shown) [Supplemental Material, Table 2 (doi:10.1289/ehp.0901559)].

Results of linear regression analyses also indicate that persons with diabetes had arsenic exposure levels similar to those without diabetes. In models adjusted for age, sex, BMI, smoking status, and educational attainment, the ratio of geometric means of arsenic exposure comparing cases and noncases was 1.00 (95%, 0.78–1.29), 0.96 (95%, 0.84–1.09), and 1.06 (95%, 0.95–1.19) for TWA, total urinary arsenic, and urinary creatinine-adjusted arsenic, respectively. In sensitivity analyses comparing diabetics with glucosuria (*n* = 141) and nondiabetics with negative urine glucose test (*n* = 10,407), ratios of geometric mean for TWA, total urinary arsenic, and urinary creatinine-adjusted arsenic were 1.10 (95% CI, 0.79–1.52), 0.91 (95% CI, 0.77–1.07), and 1.04 (95% CI, 0.91–1.20), respectively.

We observed no association between either TWA or urinary arsenic concentration and glucosuria in the overall study population (Table 3). Associations also were not evident among participants with categories of BMI levels defined by the median value. Additional adjustment for urinary creatinine did not change the estimates appreciably.

We also found no evidence of an association between arsenic exposure and HbA1c [Supplemental Material, Table 3 (doi:10.1289/ehp.0901559)]. The adjusted means of HbA1c among participants with different levels of TWA and urinary arsenic were similar. Among the 368 participants with data on both urinary arsenic metabolites and HbA1c, there was no evidence that HbA1c levels differed by %MMA, %InAs, or %DMA, nor did HbA1c levels differ by PMI or SMI. The associations were not significant in any individual categories or trend tests.

## Discussion

In this large cross-sectional study of arsenic exposure and diabetes-related outcomes, we found that arsenic exposure, measured using either TWA or urinary arsenic concentration, was not related to diabetes, glucosuria, or blood HbA1c level.

The availability of data on environmental exposure to arsenic is a strength of the present study. Unlike previous studies of lower-level arsenic exposure, which lacked reproducible and valid measures of arsenic exposure and/or information on the nature of the exposure (Lewis et al. 1999; Navas-Acien et al. 2008; Zierold et al. 2004), our study population was well described with detailed data on the duration, source, and form of exposure. The average durations of well use for wells with a known arsenic concentration were 10.0 years for men and 8.3 years for women. With chronic and continuing exposure, steady-state concentrations in blood and urine are achieved. Well-water arsenic was correlated with total urinary arsenic, urinary DMA, and urinary MMA concentration in our study population (of 0.70, 0.61, and 0.57, respectively) and was clearly the primary source of arsenic in the urine. In NHANES, urinary concentration of nontoxic AsB and total urinary arsenic were highly correlated with each other (*r* = 0.80) (Navas-Acien et al. 2008). On the contrary, consumption of seafood contributed very little to total urinary arsenic in our study population. Correlations of total urinary arsenic with urinary MMA and DMA concentrations (0.90 and 0.98, respectively) were much higher than with urinary AsB and AsC (0.13 and 0.06, respectively); AsB and AsC accounted for only 3% of total urinary arsenic in our study population.

HbA1c is a valid and reliable biomarker for long-term blood glucose level and is strongly associated with diabetes and prediabetes (Buell et al. 2007; Herman et al. 2000; Rohlfing et al. 2000). Two other studies have evaluated differences in HbA1c in relation to arsenic exposure. Hansen et al. found that HbA1c level was elevated in taxidermists and workers working with arsenic-impregnated wood (Jensen and Hansen 1998). However, the study was small, with 40 workers and 26 controls, and the levels of occupational arsenic exposure were high. In the recent NHANES analysis, Navas-Acien et al. (2008), found no association between total urinary arsenic and HbA1c.

Animal and *in vitro* model systems have indicated that arsenic exposure can potentially increase the risk of diabetes through its effects on the inhibition of insulin-dependent glucose uptake (Walton et al. 2004) and insulin signaling (Paul et al. 2007), impairment of insulin secretion and transcription in pancreatic beta cells (Diaz-Villasenor et al. 2006), and modification of the expression of genes involved in insulin resistance (Diaz-Villasenor et al. 2007). However, the concentrations used in most mechanistic experiments are high, and the observed effects may not be applicable to populations chronically exposed to arsenic in the environment. Nevertheless, the epidemiologic literature suggests that diabetes is an adverse outcome associated with prolonged exposure to high levels of water arsenic (> 500 μg/L). For instance, in a cross-sectional study of 1,595 subjects in Bangladesh, Rahman et al. (1999) reported an OR for diabetes of 1.7 (95% CI, 1.0–2.9) comparing arsenic exposure of > 10,000 μg/L-years to the unexposed group among those free of skin lesion. Among patients with skin lesions, a marker of prolonged exposure, the OR for diabetes in association with 500–1,000 μg/L and > 1,000 μg/L was 2.2 (95% CI, 1.3–3.8) and 2.6 (95% CI, 1.5–4.6), respectively (Rahman et al. 1999). In a cohort study with 41 incident cases of diabetes in southwestern Taiwan, the OR was 2.1 (95% CI, 1.1–4.2) comparing individuals with cumulative arsenic exposure > 17,000 μg/L-years to those with < 17,000 μg/L-years (Tseng et al. 2000). We did not find evidence of an association even when comparing the highest quintile of exposure (176–864 μg/L; mean, 291.2 μg/L) with the lowest (0.1–8 μg/L; mean, 2.4 μg/L). As 90% of our study population was exposed to well water arsenic < 300 μg/L, the absence of an association in this study suggests that the effects of arsenic exposure on the risk of diabetes levels between 10 and 300 μg/L are not significant. Taken together, the experimental and epidemiologic evidence suggests that the adverse effects on diabetes may be dose specific and limited to populations with prolonged exposure to very high levels of arsenic exposure.

The ratios of MMA/InAs and DMA/MMA in urine are indicative of efficiency for the first and second methylation steps, respectively, and they have been related to the risk of an array of arsenic-related health effects including the risk of skin cancer (Chen et al. 2003a; Hsueh et al. 1997; Yu et al. 2000), urothelial carcinoma (Pu et al. 2007), bladder cancer (Chen et al. 2003b), skin lesions (Ahsan et al. 2007), and hypertension (Huang et al. 2007). We found that HbA1c levels did not differ by the composition or absolute levels of urinary arsenic metabolites, which suggests that arsenic methylation capacity does not influence the risk of diabetes.

Several methodological issues should be noted in interpreting the results of our study. Similar to past reports on the topic, diabetes status was ascertained using self-report of a physician’s prior diagnosis. To the extent that diabetes status was misclassified, the association between arsenic exposure and diabetes prevalence could have been underestimated. Sensitivity analyses with cases restricted to diabetes with glucosuria did not alter associations between arsenic exposure and diabetes prevalence, which suggests that associations would not be specific to severe cases of diabetes. A continuum of risk for the development of diabetes based on HbA1c levels has been demonstrated (Droumaguet et al. 2006; Edelman et al. 2004). Thus, the lack of trend in HbA1c levels and glucosuria prevalence across categories of arsenic exposure further indicate no association with diabetes in this study. Diabetes is a complex metabolic disorder. Because individuals with diabetes may also have altered xenobiotic metabolism and excretion, the utility of using urinary arsenic concentrations as the biomarker of exposure could be limited (Kile and Christiani 2008). Steinmaus et al. (2009a, 2009b) suggested that creatinine adjustment may be one of the reasons for the strong association observed in NHANES data. Analogously, Gamble et al. have reported that correlation between urinary arsenic and plasma folate may due in part to the correlation between folate and urinary creatinine (Gamble and Liu 2005). Given the potential limitation in the utility of urinary creatinine in retrospective case–control and cross-sectional studies, further prospective studies would be valuable. Finally, our study population in general was lean with regard to low socioeconomic and nutritional status. Thus, the findings may not be generalizable to other study populations, given the possible different distribution of risk factors for diabetes that may influence the effect of arsenic exposure.

In conclusion, we did not find an association between arsenic exposure from drinking water and diabetes-related outcomes. Although it is important to minimize arsenic exposure because of the increased risk of cancer and many other health effects, our study indicates that arsenic exposure, in the range of levels observed, does not pose a significant risk for diabetes.

## Figures and Tables

**Table 1:** Distribution of demographic, lifestyle, and arsenic exposure variables by diabetes status.

	Diabetes		
Variable^a^	Yes (*n* = 241)	No (*n* = 11,078)	*p*-Value^b^
Men [*n* (%)]	120 (49.8)	4,736 (42.8)	0.03
Age (years)	42 (30, 56)	36 (25, 50)	< 0.01
Education attainment (years)	5 (0, 10)	2 (0, 10)	< 0.01
TV ownership [*n* (%)]	124 (51.5)	3,746 (33.8)	< 0.01
Land ownership [*n* (%)]	161 (67.1)	5,424 (49.0)	< 0.01
Cigarette-smoking status [*n* (%)]			
Never	157 (65.2)	7,152 (64.6)	< 0.01
Past	29 (12.0)	717 (6.5)	
Current	55 (22.8)	3,209 (28.9)	
Ever users of betel nut [*n* (%)]	111 (46.1)	4,220 (37.4)	0.01
Dietary intake of rice (g/day)	1,461 (519, 2,070)	1,554 (1,035, 2,101)	< 0.01
Dietary intake of fish (g/day)	50 (21, 99)	47 (17, 98)	0.20
Systolic blood pressure (mmHg)	122 (100, 151)	112 (95, 136)	< 0.01
Diastolic blood pressure (mmHg)	80 (65, 97)	73 (60, 89)	0.02
BMI (kg/m^2^)	22.5 (17.6, 26.7)	19.2 (16.3, 24.0)	< 0.01
≥ 20 [*n* (%)]	54 (22.9)	5,467 (50.5)	< 0.01
< 20 [*n* (%)]	182 (77.1)	5,356 (49.5)	
Urinary creatinine (mg/dL)^c^	44.1 (15.9, 102.2)	45.9 (14.6, 120.1)	0.03
Arsenic concentration of index well (μg/L)	53.0 (1.2, 236.0)	62.0 (1.3, 264.0)	0.08
TWA (μg/L)	53.0 (1.3, 258.0)	62.0 (1.7, 255.2)	0.33
0.1–8.0 [*n* (%)]	52 (22.0)	2,206 (20.6)	0.50
8.1–41.0 [*n* (%)]	53 (22.5)	2,098 (19.6)	
41.2–91.7 [*n* (%)]	49 (20.8)	2,102 (19.7)	
91.8–176.1 [*n* (%)]	38 (16.1)	2,151 (20.1)	
176.2–864.0 [*n* (%)]	45 (18.6)	2,140 (20.0)	
Urinary arsenic concentration (μg/L)^c^	72.0 (23, 274)	87.0 (23, 311)	0.08
1–36	50 (21.5)	2,160 (20.4)	
37–66	58 (24.9)	2,071 (19.6)	
67–114	46 (19.7)	2,126 (20.1)	
115–204	43 (18.5)	2,110 (19.9)	
≥ 205	36 (15.5)	2,126 (20.1)	
Creatinine-adjusted urinary arsenic (μg/g creatinine)^c^	175.7 (66.5, 536.8)	200.0 (62.5, 598.9)	< 0.01
Urinary glucose (mg/dL)^d^ [*n* (%)]			
Negative	90 (39.0)	10,407 (99.1)	< 0.01
50	23 (10.0)	39 (0.4)	
100	14 (6.1)	16 (0.2)	
≥ 200	104 (44.9)	35 (0.3)	
HbA1c (%)^e^	6.8 (4.7, 10.9)	4.9 (4.4, 5.4)	< 0.01

Abbreviations: DBP, diastolic blood pressure; SBP, systolic blood pressure. Values shown are median (10th percentile, 90th percentile) except where indicated.

a Data were missing for the following variables: BMI (5 cases and 255 noncases); education (0 and 6 subjects); TV ownership (0 and 2 subjects); rice intake (0 and 18 subjects); fish intake (5 and 131 subjects); blood pressure (2 and 237 subjects); and TWA (5 and 381 subjects).

b *p*-Values from the chi-square test or *t*-test.

c Based on 233 cases and 10,593 noncases with urine samples and total urinary arsenic analysis results.

d Based on 231 cases and 10,497 noncases with urine samples and urine glucose dipstick test results that were available.

e Based on a subgroup of 45 diabetes cases and 1,999 noncases.

**Table 2:** Associations [OR (95% CI)] between arsenic exposure and diabetes.

	Quintiles					
Arsenic exposure variable	1	2	3	4	5	*p* for trend^a^
TWA (μg/L)	0.1–8.0	8.1–41.0	41.2–91.7	91.8–176.1	176.2–864.0	

*n* (cases/noncases)	52/2,206	53/2,098	49/2,102	38/2,151	44/2,140	
Model 1^b^	1.00	1.28 (0.85–1.91)	1.20 (0.80–1.81)	0.95 (0.61–1.47)	1.08 (0.71–1.65)	0.95
Model 2^c^	1.00	1.35 (0.90–2.02)	1.24 (0.82–1.87)	0.96 (0.62–1.49)	1.11 (0.73–1.69)	0.33

Urinary arsenic (μg/L)	1–36	37–66	67–114	115–204	≥ 205	

*n* (cases/noncases)	50/2,160	58/2,071	46/2,126	43/2,110	36/2,126	
Model 1^b^	1.00	1.29 (0.87–1.91)	0.99 (0.65–1.50)	0.90 (0.59–1.39)	0.87 (0.56–1.36)	0.09
Model 2^c^	1.00	1.29 (0.87–1.91)	1.05 (0.69–1.59)	0.94 (0.61–1.44)	0.93 (0.59–1.45)	0.14
Model 3^d^	1.00	1.44 (0.97–2.17)	1.20 (0.77–1.85)	1.16 (0.73–1.85)	1.22 (0.73–2.03)	0.83

High BMI (BMI ≥ 20)^e^						
TWA (μg/L)						
*n* (cases/noncases)	39/883	32/803	32/805	27/797	35/740	
Model 2^c^	1.00	1.02 (0.63–1.67)	1.01 (0.62–1.65)	0.86 (0.51–1.42)	1.13 (0.70–1.82)	0.72

Urinary arsenic (μg/L)						
*n* (cases/noncases)	35/879	39/850	31/824	35/803	26/729	
Model 2^c^	1.00	1.16 (0.72–1.87)	1.01 (0.61–1.68)	1.14 (0.70–1.87)	1.06 (0.62–1.80)	0.70
Model 3^d^	1.00	1.35 (0.83–2.21)	1.17 (0.69–1.98)	1.46 (0.85–2.51)	1.41 (0.77–2.59)	0.41

Low BMI (BMI < 20)^e^						
TWA (μg/L)						
*n* (cases/noncases)	13/1,323	21/1,295	17/1,297	11/1,354	9/1,400	
Model 2^c^	1.00	1.74 (0.86–3.49)	1.35 (0.65–2.79)	0.83 (0.37–1.87)	0.66 (0.28–1.56)	0.07

Urinary arsenic (μg/L)						
*n* (cases/noncases)	15/1,281	19/1,221	15/1,302	8/1,307	10/1,397	
Model 2^c^	1.00	1.53 (0.75–3.12)	1.11 (0.52–2.34)	0.51 (0.20–1.27)	0.70 (0.30–1.60)	0.07
Model 3^d^	1.00	1.62 (0.79–3.34)	1.23 (0.56–2.69)	0.59 (0.22–1.55)	0.87 (0.34–2.25)	0.15

a Estimated using arsenic exposure as a continuous variable in the model.

b Model 1: ORs were adjusted for age, sex, and BMI.

c Model 2: ORs were adjusted for age, sex, BMI, smoking status, and educational attainment.

d Model 3: ORs were adjusted for age, sex, BMI, smoking status, educational attainment, and urinary creatinine.

e *p*-Values for interaction between arsenic exposure and BMI were 0.76 (for well water arsenic, Model 2), 0.90 (for urinary arsenic, Model 2), and 0.89 (for urinary arsenic, Model 3).

**Table 3:** Associations [OR (95% CI)] between arsenic exposure and glucosuria.^a^

	Quintiles					
Arsenic exposure variables	1	2	3	4	5	*p* for trend^b^
TWA (μg/L)	0.1–8.0	8.1–41.0	41.2–91.7	91.8–176.1	176.2–864.0	

*n* (cases/noncases)	52/2,099	44/2,015	44/1,961	41/1,983	48/1,985	
Model 1^c^	1.00	1.02 (0.67–1.55)	1.09 (0.72–1.65)	1.01 (0.66–1.54)	1.18 (0.78–1.77)	0.28
Model 2^d^	1.00	1.07 (0.71–1.63)	1.12 (0.74–1.71)	1.01 (0.66–1.56)	1.20 (0.79–1.81)	0.30

Urinary arsenic (μg/L)	1–36	37–66	67–114	115–204	≥ 205	

*n* (cases/noncases)	59/2,118	50/2,032	42/2,088	37/2,079	42/2,074	
Model 1^c^	1.00	0.90 (0.61–1.34)	0.74 (0.49–1.12)	0.63 (0.41–0.97)	0.84 (0.56–1.27)	0.31
Model 2^d^	1.00	0.91 (0.62–1.34)	0.79 (0.52–1.19)	0.65 (0.43–1.01)	0.91 (0.60–1.37)	0.46
Model 3^e^	1.00	0.95 (0.63–1.42)	0.85 (0.55–1.31)	0.72 (0.45–1.15)	1.03 (0.63–1.68)	0.90

High BMI (BMI ≥ 20)						
TWA (μg/L)						
*n* (cases/noncases)	43/849	28/779	29/764	32/743	35/694	
Model 2^d^	1.00	0.78 (0.45–1.38)	0.83 (0.51–1.35)	0.93 (0.58–1.50)	1.03 (0.65–1.65)	0.60

Urinary arsenic (μg/L)						
*n* (cases/noncases)	46/855	37/828	26/804	30/790	30/703	
Model 2^d^	1.00	0.80 (0.51–1.27)	0.60 (0.36–1.01)	0.67 (0.41–1.08)	0.87 (0.54–1.41)	0.50
Model 3^e^	1.00	0.84 (0.53–1.33)	0.64 (0.38–1.08)	0.73 (0.43–1.24)	0.96 (0.55–1.69)	0.87

Low BMI (BMI < 20)						
TWA (μg/L)						
*n* (cases/noncases)	9/1,250	16/1,236	15/1,197	9/1,240	13/1,291	
Model 2^d^	1.00	1.93 (0.85–4.39)	1.82 (0.79–4.18)	1.03 (0.40–2.61)	1.45 (0.62–3.42)	0.82

Urinary arsenic (μg/L)						
*n* (cases/noncases)	13/1,263	13/1,204	16/1,284	7/1,289	12/1,371	
Model 2^d^	1.00	1.03 (0.47–2.23)	1.19 (0.57–2.50)	0.50 (0.20–1.26)	0.88 (0.40–1.94)	0.54
Model 3^e^	1.00	1.00 (0.53–2.49)	1.15 (0.53–2.49)	0.47 (0.18–1.26)	0.82 (0.33–2.04)	0.54

a Because treatments for diabetes may influence glucosuria status, participants with diabetes were excluded from those who tested negative for glucosuria in estimating ORs for glucosuria (*n* = 90).

b Estimated using arsenic exposure as a continuous variable in the model.

c Model 1: ORs were adjusted for age, sex, and BMI.

d Model 2: ORs were adjusted for age, sex, BMI, smoking status, and educational attainment.

e Model 3: ORs were adjusted for age, sex, BMI, smoking status, educational attainment, and urinary creatinine.
